# How pressure enhances the critical temperature of superconductivity in YBa_2_Cu_3_O_6+*y*_

**DOI:** 10.1073/pnas.2215458120

**Published:** 2023-01-06

**Authors:** Michael Jurkutat, Carsten Kattinger, Stefan Tsankov, Richard Reznicek, Andreas Erb, Jürgen Haase

**Affiliations:** ^a^Felix Bloch Institute for Solid State Physics, Leipzig University 04103, Leipzig, Germany; ^b^Walther Meissner Institut, Bayerische Akademie der Wissenschaften 85748, Garching, Germany

**Keywords:** superconductivity, pressure, NMR

## Abstract

High-temperature superconducting cuprates respond to doping with a dome-like dependence of their critical temperature (*T*_c_). But the family-specific maximum *T*_c_ can be surpassed by application of pressure, a compelling observation known for decades. We investigate the phenomenon with high-pressure anvil cell NMR and measure the charge content at planar Cu and O, and with it the doping of the ubiquitous CuO_2_ plane with atomic-scale resolution. We find that pressure increases the overall hole doping, as widely assumed, but when it enhances *T*_c_ above what can be achieved by doping, pressure leads to a hole redistribution favoring planar O. This is similar to the observation that the family-specific maximum *T*_c_ is higher for materials where the hole content at planar O is higher at the expense of that at planar Cu. The latter reflects dependence of the maximum *T*_c_ on the Cu–O bond covalence and the charge-transfer gap. The results presented here indicate that the pressure-induced enhancement of the maximum *T*_c_ points to the same mechanism.

## Significance

Understanding cuprate superconductivity is still at the center of condensed matter physics, and the fact that external pressure can enhance the maximum temperature of superconductivity *T*_c, max_ in excess of what can be achieved by chemistry has puzzled researchers for decades. Using high-pressure NMR, we quantify the charge distribution within the Cu–O bonds of these materials and find that pressure can lead to a redistribution of charge between both atoms and thereby increase *T*_c, max_. This is in line with previous NMR analyses at ambient pressure that revealed that *T*_c, max_ of the different cuprate families appears to be set by this distribution, as well, which reflects the crucial role of the charge-transfer gap or the Cu–O bond covalence.

High-temperature superconducting cuprates (1) are still central to condensed matter physics, and they carry surprisingly rich electronic properties (2) despite sharing a rather simple CuO_2_ plane as a common structural unit. By doping the antiferromagnetic parent materials with electrons or holes, these properties are induced, in particular, superconductivity with its critical temperature (*T*_c_) that shows a dome-like dependence on the doping level. However, while the maximum value *T*_c, max_ appears at the so-called optimal doping levels (near ∼16%) for all materials, the family-dependent *T*_c, max_ differ widely.

Previously, it was shown that nuclear magnetic resonance (NMR) can measure the charges in the CuO_2_ plane at the atomic level, i.e., in terms of the Cu (*n*_Cu_) and O (*n*_O_) bonding orbital hole contents, and a simple relation was found (3),

[1] $$\begin{array}{c} 1+\zeta =n_{\mathrm{Cu}}+2n_{O}. \end{array}$$

**Fig. 1.**
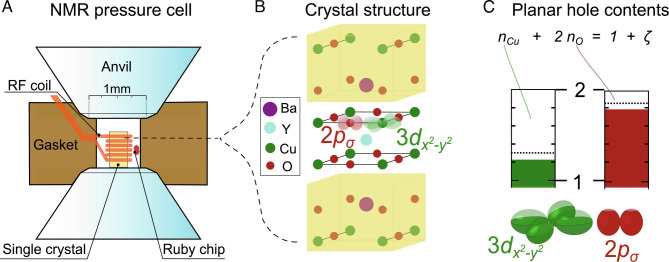
Anvil cell high-pressure exerted on YBa_2_Cu_3_O_6 + *y*_ changes the charges in the CuO_2_ plane. (*A*) Schematic of the anvil cell used for NMR; the microcoil surrounds the single crystal of about 1 nano-L volume, and both are placed in the high-pressure chamber with a ruby chip as an optical pressure gauge. (*B*) Sketch of the crystal structure of YBa_2_Cu_3_O_6 + *y*_ with highlighted bonding orbitals in one of the CuO_2_ planes. (*C*) The hole content of these bonding orbitals can be measured with Cu and O NMR quadrupole splittings; see *Methods*. From the measured hole contents for Cu (*n*_Cu_) and O (*n*_O_), the total doping measured by NMR, *ζ*, follows (1 + *ζ* = *n*_Cu_ + 2*n*_O_).

**Fig. 2.**
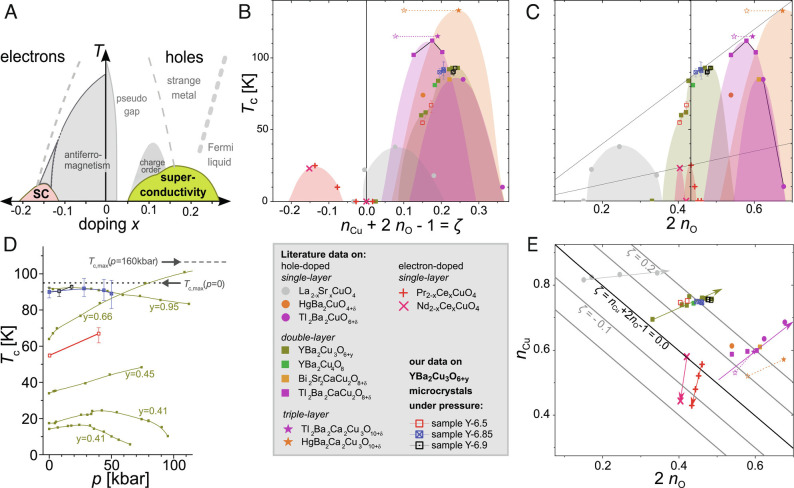
Charges in the CuO_2_ plane and *T*_c_. (*A*) An electronic phase diagram is typically used to mark the various cuprate phenomena as a function of doping (*x*) and temperature (*T*). However, important details like the maximum *T*_c_ differ between material families and are not set by *x*. Also, materials differ in the mode of chemical alteration to dope the CuO_2_-plane, indicated in the stoichiometries in the legend; for details, see *Materials and Methods*. (*B*) *T*_c_ as a function of *ζ* = *n*_Cu_ + 2*n*_O_ − 1, i.e., the doping measured with NMR. (*C*) *T*_c_ as a function of 2*n*_O_ orders the superconducting domes; a near proportionality between *T*_c, max_ and *n*_O_ is revealed (4). (*D*) *T*_c_ vs. pressure for different doping levels of YBa_2_Cu_3_O_6 + *y*_ (YBCO) from literature data (10) and for the samples measured here, cf. legend. *T*_c_ slowly decreases for optimally doped YBCO with pressure, while *T*_c_ increases for underdoped YBCO and can even exceed the maximum *T*_c_ achievable with chemical doping (gray lines). Results from this work are shown for three different materials together with literature data in panels *B*–*E*, cf. legend. (*E*) The planar charge distribution in terms of *n*_Cu_ and 2*n*_O_ (3) reveals significant differences between the various families. This display relates to the above panels, sharing the abscissa with *C* and indicating diagonal lines of constant doping *ζ* corresponding to the abscissa of *B*.

This relation is expected if *ζ* is similar to the chemical doping that adds to the hole already present in the parent compound, where Cu is nominally in 3d^9^ configuration. Interestingly, it was found that the actual sharing of the hole content between Cu and O appears to be a fundamental parameter for *T*_c_: *T*_c, max_ is nearly proportional to *n*_O_ (4), which is largely determined by the parent chemistry. So to increase *T*_c, max_, electron charge has to be transferred from planar O to planar Cu, an experimental correlation that holds for all known hole-doped cuprates, *T*_c, max_ ≈ 200K ⋅ 2*n*_O_ (4), cf. also Figs. 1 *B* and *C* and 2*A*–*C* and *E*. This identifies the material chemistry parameter controlling the hole sharing between Cu and O—the charge-transfer gap or bond covalence^*^—as crucial in determining the maximum *T*_c_ obtainable at optimal doping. An observation that only recently could be reproduced theoretically by solving the three-band Hubbard model (5). Experimentally, O’Mahony et al. (6) provided evidence that the charge-transfer energy locally correlates with the superconducting electron-pair condensate.

The intriguing relation between *T*_c, max_ and the hole sharing in the CuO_2_ plane may hold information about another mystery of cuprate behavior: the unusual, material-specific pressure dependence of *T*_c_ (7, 8). While the pressure response of *T*_c_ is different for the various cuprate families, quite generally, *T*_c_ of underdoped cuprates tends to increase with pressure (*p*), while it hardly changes for optimal doping and usually decreases for overdoped materials (9, 10). This suggests that pressure increases planar hole doping, which is also supported by conductivity and Hall measurements (7–9, 11). However, some underdoped samples show a significantly higher *T*_c, max_(*p*) than what can be achieved by chemical doping (*x*), as is the case for YBa_2_Cu_3_O_6 + *y*_ investigated here, cf. Fig. 2*D*. This phenomenon is of great interest as it may relate to the mechanism of superconductivity.

Clearly, with the NMR results mentioned above, it appears intriguing to explore how pressure affects the charges in the CuO_2_ plane at the atomic level. However, since this requires ^63^Cu and ^17^O NMR experiments on oriented single crystals at pressures that can only be achieved with anvil cell devices, such experiments are rather challenging: A microcrystal surrounded by a radio frequency microcoil needs to be positioned inside the pressurized region of an anvil cell, as depicted in Fig. 1*A*. Apart from mechanical issues such as disruptive failures induced by a changing geometry with pressure, for example, the signal-to-noise for the aligning process of the microcrystal with respect to the magnetic field, necessary for every pressure point of every sample, is a limiting factor. We were able to meet these requirements to some extent and report on the results obtained for a few microcrystals of YBa_2_Cu_3_O_6 + *y*_ at pressures up to about 44kbar. We find that pressure indeed has two effects: it increases the overall hole doping in the plane, as is generally expected, but it also changes the sharing of the holes between Cu and O and can thereby increase *T*_c_ as well. These findings underline the importance of the sharing of the planar charges for *T*_c, max_ and the therein reflected role of the charge-transfer gap and the Cu–O bond covalence (5).

In this manuscript, we focus on the relationship between the maximum *T*_c_ and average local charge on Cu and O measured by NMR, and we will not address the rich, doping-dependent cuprate phenomena (2) including intraunit-cell order (12–14).

## 1. Planar Charge Distribution and T_c_ Under Pressure

The common electronic phase diagram of the cuprates, cf. Fig. 2*A*, assumes that chemical doping (*x*) is the key variable. Since we can measure the NMR doping level *ζ* given by **Eq. 1**, we prefer to use *ζ* as the actual doping level, if NMR measurements are available. To keep the discussion transparent, we use the variable *x* if chemical doping is known from other sources, e.g., by using the superconducting dome or from stoichiometry. Slight differences between the two numbers (*ζ* and *x*) become apparent by noting that the superconducting domes do not fall exactly on top of each other in a *T*-*ζ* phase diagram, cf. Fig. 2*B*.

The sharing of charges between Cu and O in the CuO_2_ plane (at ambient conditions) is reproduced in Fig. 2*E* (3), where the black diagonal “parent” line (*ζ* = 0) separates the hole-doped regime above (*ζ* >  0) from the electron-doped below it (*ζ* <  0). The various cuprate families then start at very different points near the parent line. These starting points also determine the ratio (*Δn*_Cu_/2*Δn*_O_) of how the doped holes entering the plane are distributed (slopes indicated by arrows). This is because sharing of both inherent and doped charges is determined by the material-dependent planar bond covalence.

**Fig. 3.**
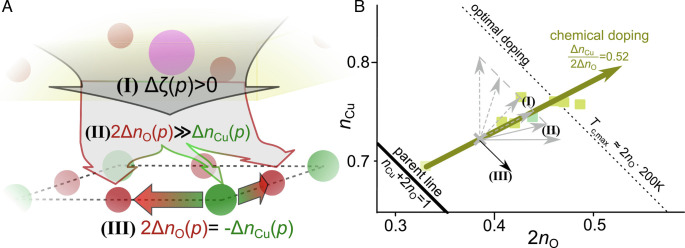
Pressure effects on planar charges. (*A*) Pressure can induce hole doping of the CuO_2_ plane (I), and the holes arrive predominantly at planar O (II), but pressure can also induce intraplanar hole redistribution from Cu to O (III). (*B*) The same effects are described in the “YBCO-region” of the *n*_Cu_ − 2*n*_O_ plane from Fig. 2*E*, with the parent line indicated in black. An underdoped (*x* ∼ 10%) YBCO system would be located near (*n*_Cu_, 2*n*_O_) = (0.72,0.38), indicated by a gray cross. If pressure (I) increases hole doping to a certain level (dashed line parallel to the optimal doping line), the system could follow any of the gray arrows. If (II) pressure favors hole doping of planar O more than chemical doping, a shallower slope is expected (full gray arrows). (III) If the charges are redistributed within the plane, the system would follow the black full arrow given the overall doping remained the same.

We now focus on YBa_2_Cu_3_O_6 + *y*_ (YBCO), full dark yellow squares in Fig. 2*E* or in more detail in Fig. 3*B*. In the undoped (*y* = 0) material YBa_2_Cu_3_O_6_, the inherent hole must be shared between Cu and O, and we estimate *n*_Cu_ = 0.68 and 2*n*_O_ = 0.32 from this plot. Upon doping, the holes enter the CuO_2_ plane as indicated by the dark yellow arrow that points away from the parent line with the slope of *Δn*_Cu_/2*Δn*_O_ ≈ 0.52, cf. Fig. 3*B*. The question this work aims to address is how pressure affects the planar charges *n*_Cu_ and *n*_O_.

In Fig. 3, we show schematically what could be expected if pressure enhances *T*_c_ by increasing the O hole content. Given the general pressure dependence of *T*_c_ as well as conductivity and Hall measurements (7–9, 11), one expects that (I) pressure increases planar hole content, *Δ*_*p*_*ζ* >  0. (II) To increase the maximum *T*_c_ by boosting the O hole content under pressure, clearly, pressure-induced hole-doping should favor O more than chemical doping does, i.e., *Δ*_*p*_*n*_Cu_/2*Δ*_*p*_*n*_O_ <  0.52. This would require a decrease in the charge-transfer gap and an increase in bond covalence, which would (III) cause an intraplanar hole-transfer from Cu to O, although this effect could be masked by coincident pressure-induced doping.

In the literature (15), the change of *T*_c_ with pressure is typically described phenomenologically as:

[2] $$\begin{array}{c} {\left(\frac{dT_{c}}{\mathrm{dp}}\right)}_{\mathrm{tot}}=\left(\frac{\partial T_{c}}{\partial x}\right)\left(\frac{\partial x}{\partial p}\right)+{\left(\frac{dT_{c}}{\mathrm{dp}}\right)}_{\mathrm{intr}}. \end{array}$$

The first term on the r.h.s. describes the change of doping due to pressure (∂*x*/∂*p*) with ∂*T*_c_/∂*x* given by the slope of the superconducting dome as a function of doping at ambient pressure. The second term (*dT*_c_/*dp*)_intr_ describes the (unknown) intrinsic pressure effects on *T*_c_, i.e., pressure-induced change of the shape of the superconducting dome. Although **Eq. 2** is not necessary for our analysis, we will discuss our results also in this context.

## 2. High-Pressure NMR Experiments

In order to measure the planar charges under pressure, high-pressure ^63^Cu and ^17^O anvil cell NMR experiments were performed with homemade anvil cells (16) that fit standard NMR magnets (11.7T and 17.6T) and homemade probes. Therefore, our anvil cells are rather small compared to what is used by another group (17) that also engages in single crystal NMR experiments (of other materials) at similar pressures. We use ^17^O exchanged small-volume (0.3 to 1.5 nano-L) microcrystals with three different stoichiometries: YBa_2_Cu_3_O_6.5_ (Y-6.5), YBa_2_Cu_3_O_6.85_ (Y-6.85), and YBa_2_Cu_3_O_6.9_ (Y-6.9). These doping levels were originally determined from *T*_c_ measurements (see *SI Appendix*, section 3). The crystals were glued on one of the anvil’s culets, and radio frequency (RF) microcoils were placed around them with the leads fed to outside the pressurized region through channels carved in the gasket; paraffin oil ensured hydrostatic conditions. Pressure was applied with a hydraulic press, and screws secured the pressure during NMR experiments.

Standard orientation-dependent NMR experiments were performed to measure the quadrupole frequencies (splitting of the Zeeman resonance) for ^63^Cu and ^17^O in the CuO_2_ plane, from which the hole densities can be determined (see *Methods*). The NMR doping levels at ambient pressure for the samples used here are *ζ* = 0.15, 0.19, and 0.23 for Y-6.5, Y-6.85, and Y-6.9, respectively.

**Fig. 4.**
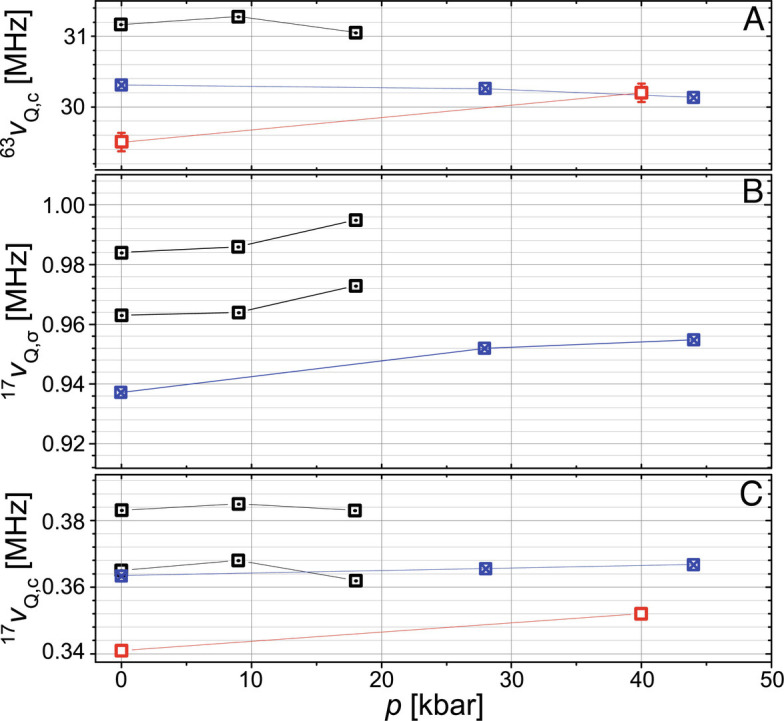
Experimental data. Pressure dependence of the ^17^O and ^63^Cu quadrupole frequencies (^17, 63^*ν*_*Q*, *α*_) for the aligned single crystals of Y-6.9 (dotted black squares), Y-6.85 (crossed blue squares), and Y-6.5 (red open squares); *α* denotes the direction of the external magnetic field *B*_0_. (*A*) ^63^Cu along the crystal *c*-axis, (*B*) ^17^O along the *σ*-bond direction, and (*C*) ^17^O along the crystal *c*-axis. For Y-6.9, both quadrupole frequencies reflecting the double peak feature of the satellite transitions are displayed (the error, indicated, is typically much less than the symbol size).

The measured pressure dependence of the NMR quadrupole frequencies (${{}^{63,17}\nu }_{Q}$) of the aligned single crystals is summarized in Fig. 4. For planar Cu in Fig. 4*A*, we find that ^63^*ν*_Q, c_ increases for the underdoped Y-6.5, but it is less sensitive to pressure in the higher doped Y-6.85 and Y-6.9 and even slightly decreases at elevated pressure. Both observations are consistent with previous Cu NMR reports on underdoped and optimally doped YBCO (18).

For planar O in Fig. 4 *B* and *C*, we find that ^17^*ν*_Q, c_ (field along the crystal *c*-direction) and ^17^*ν*_Q, σ_ (field along the Cu–O *σ*-bond) generally increase with pressure for all doping levels, although this is more pronounced for the underdoped Y-6.5. While the literature on ^17^O NMR in cuprates under pressure is limited, one study on single crystals of underdoped YBCO up to 18 kbar found increasing ^17^O quadrupole splittings as well (19). We note that the peculiar changes in splittings for Y-6.9 have been shown to signify charge ordering in that compound at elevated pressure (20).

Using the pressure-induced changes of the ^63^Cu and ^17^O NMR quadrupole splittings depicted in Fig. 4, we determined the planar charges as a function of pressure, see Eqs. **3** and **4** in *Materials and Methods*.

## 3. Planar Charges Under Pressure

**Fig. 5.**
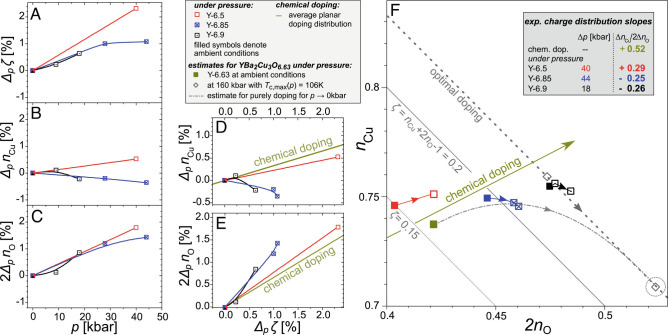
Planar charge distribution as determined by NMR for samples Y-6.9 (black), Y-6.85 (blue), and Y-6.5 (red). (*A*) All samples show increasing hole-doping *Δ*_*p*_*ζ* = *Δ*_*p*_*n*_Cu_ + 2*Δ*_*p*_*n*_O_ >  0 with pressure. The slope *Δ*_*p*_*ζ*/*Δp* is higher in underdoped Y-6.5. (*B*) The Cu hole content is found to increase for the underdoped sample Y-6.5, *Δ*_*p*_*n*_Cu_ >  0; this is weaker for higher doping and *n*_Cu_ even decreases at elevated pressure. (*C*) The O hole content 2*n*_O_ increases for all samples similarly, with approximately 4 × 10^−4^ holes/kbar. In order to compare pressure effects to chemical doping (dark yellow lines), we show *Δ*_*p*_*n*_Cu_ and 2*Δ*_*p*_*n*_O_ as a function of pressure-induced doping *Δ*_*p*_*ζ* in (*D*) and (*E*), respectively. The increase in Cu hole content, *Δ*_*p*_*n*_Cu_, is smaller for pressure-induced doping compared with that induced by chemical doping for all samples. The O hole content 2*Δ*_*p*_*n*_O_ increases much faster with pressure-induced doping compared to chemical doping for all samples. For both Cu and O, the underdoped Y-6.5 is closest to chemical doping, where the higher doped Y-6.9 and Y-6.85 also show an intraplanar charge redistribution, i.e., an increase of O holes at the expense of Cu holes. (*F*) Zoom into the (*n*_Cu_, 2*n*_O_)-plane (cf. Figs. 2*E* and 3*B*) near our experimental data (full symbols denote ambient pressure data, and full lines with arrows indicate increasing pressure). Also shown are literature data for YBa_2_Cu_3_O_6.63_, and the estimated high-pressure point (160 kbar, *T*_c_ = 106 K, circled empty diamond) is that of an optimally doped YBCO with appropriate charge redistribution for the enhanced *T*_c_ (*ΔT*_c, max_ = 11K, 2*Δn*_O_ ≈ 5.5%). The dash-dotted gray line is a parabolic fit between the two points, *Δ*_*p*_*n*_Cu_ = 0.52 ⋅ 2*Δ*_*p*_*n*_O_ − 8 ⋅ (2*Δ*_*p*_*n*_O_)^2^, where the linear slope 0.52 is defined by the chemical doping.

The pressure-induced changes (*Δ*_*p*_) in the average local hole contents (*Δ*_*p*_*n*_Cu_ and *Δ*_*p*_*n*_O_) add up to the total change in hole content (*Δ*_*p*_*ζ*) of the CuO_2_ plane, cf. **Eq. 1**. We find *Δ*_*p*_*ζ* >  0 for all samples, i.e., we observe an increase in hole doping with increasing pressure, cf. Fig. 5*A*. This hole doping is more pronounced for underdoped Y-6.5 with an initial slope of ≈ <  *SPSDOUBLEDOLLAR* >  5.8 × 10^−4^ holes/kbar, compared to only ≈ <  *SPSDOUBLEDOLLAR* >  3.5 × 10^−4^ holes/kbar for near optimally doped Y-6.85 as well as Y-6.9.

However, the changes of the site-specific hole contents with pressure differ between materials, as can be seen in Fig. 5 *B* and *C*. While *Δ*_*p*_*n*_Cu_/*p* ≈1.3 × 10^−4^holes/kbar for underdoped Y-6.5, the materials closer to optimal doping, Y-6.85 and Y-6.9, show a much weaker or no increase at lower pressure and even a decrease in Cu hole content beyond 10kbar, cf. Fig. 5*B*. For O, we find that pressure causes a similar increase for all three samples, i.e., 2*Δ*_*p*_*n*_O_/*p* ≈ 4 × 10^−4^holes/kbar, cf. Fig. 5*C*. This clearly indicates a pressure-induced intraplanar charge redistribution, certainly for the higher doped Y-6.85 and Y-6.9, where the O hole content increases stronger than doping, i.e., 2*Δ*_*p*_*n*_O_ >  *Δ*_*p*_*ζ*, and the Cu hole content decreases *Δ*_*p*_*n*_Cu_ <  0.

In order to compare pressure effects to chemical doping, the pressure-induced changes of the Cu and O hole contents as a function of pressure-induced doping (*Δ*_*p*_*ζ*) are shown in Fig. 5 *D* and *E*. We observe a relative decrease of *n*_Cu_ and an increase of *n*_O_, compared to what is found for chemically doped charges.

To summarize, our high-pressure NMR experiments on YBCO have shown that the increase in *T*_c_ with pressure is accompanied by changes in the local hole contents that lead to an increased *n*_O_ compared to chemical doping. All three effects displayed in Fig. 3 were observed: (I) an increase in hole doping, *Δ*_*p*_*ζ* >  0, that (II) favors an increase in O holes (*n*_O_) over those at Cu (*n*_Cu_). And at high doping levels and elevated pressure, even (III) intraplanar charge redistribution can be observed.

Clearly, the data qualitatively indicate that pressure induces hole doping as well as an increase in bond covalence. Before discussing our results more broadly, we first consider whether the observed changes in planar charges are quantitatively sufficient to account for the pressure-enhanced maximum *T*_c_ reported for YBCO as shown in Fig. 2*D*. Unfortunately, YBa_2_Cu_3_O_6.63_, which shows the highest pressure-induced increase in *T*_c_, is not part of our final set of samples. In addition, we are lacking data for much higher pressures with our single crystal anvil NMR. However, from our samples with doping levels below and above that of YBa_2_Cu_3_O_6.63_, we can nonetheless obtain a quantitative estimate. The literature data for YBa_2_Cu_3_O_6.63_ show that *T*_c_ increases from about 64K at ambient pressure to about 106K at 160 kbar. This means an increase of *T*_c, max_ of about 11K compared to that of the optimally doped material. According to the experimental relation, *T*_c, max_ ≈ 200K ⋅ 2*n*_O_, this requires an increase of 2*n*_O_ by 5.5% for an optimally doped YBCO. We can find the position of such a material in the (*n*_Cu_, 2*n*_O_)-plot by following a line of constant doping, beginning at chemically optimally doped YBCO, until we reach the encircled, empty diamond in the lower right corner of Fig. 5*F*. Applying pressure means that the hole contents move from (*n*_Cu_, 2*n*_O_) = (0.738, 0.423) to (0.709, 0.524), cf. Fig. 5*F*. So, under pressure of 160 kbar, the O hole content in YBa_2_Cu_3_O_6.63_ has to increase by 10%, i.e., at a rate of 2*Δ*_*p*_*n*_O_/*p* ≈ 6.3 × 10^−4^ holes/kbar, which is comparable to the average increase in O hole content we see for our samples of 4 × 10^−4^ holes/kbar. While we do not have data on YBa_2_Cu_3_O_6.63_ and only reach one fourth of the necessary pressure to unlock its *T*_c_(*p*) peak, we do show a possible path for illustration purposes. To reproduce the chemical doping-like hole distribution for lower doping and lower pressure, we assume a parabolic dependence that leads to the empty diamond and has an initial slope given by the chemical doping. We obtain the dash-dotted line in Fig. 5*F*, which reproduces the overall features of our experimental data quite well.

## 4. Discussion

Pressure-induced doping clearly depends on the material and chemical doping and previous assessments range widely with maximum values up to 0.2%/kbar (21, 22), while we find that ∂*ζ*/∂*p* ≈ 0.058(5)%/kbar for the underdoped Y-6.5, and 0.036(5)%/kbar for the samples near optimal doping. A recent estimate by Alireza et al. (23) of 0.032%/kbar for fully doped YBCO matches our results quite well. Note that our data imply pressure-induced doping, ∂*ζ*/∂*p*, that is stronger for underdoped YBCO, contrary to modeling assumptions used elsewhere (19, 24).

Pressure favors a higher O hole content 2*n*_O_ compared to what can be achieved by chemical doping to the extent that, particularly at higher pressure and for higher doping levels, 2*n*_O_ increases not only through doping but also at the expense of a decreasing Cu hole content (*n*_Cu_). A similar effect was recently reported with first principle calculations for Bi-based cuprates by Deng et al. (25). Their results for a pressure-induced increase of Cu 3*d*(*x*^2^ − *y*^2^) occupation and so a decrease in Cu hole content of ∂*n*_Cu_/∂*p* = −0.04%/kbar are more pronounced than what we find, cf. Fig. 5*B*. The pressure-induced decrease in *n*_Cu_, while simultaneously overall doping increases, clearly reveals an intraplanar charge redistribution under pressure.

The sharing of the inherent hole that is nominally on Cu and the distribution of additional (chemically) doped charges, both reveal Cu and O contributions to occupied and unoccupied electronic states. Depending on the context in which cuprates are discussed, this reflects the Cu–O bond covalence, the charge-transfer gap, or Cu and O band contributions. An intraplanar redistribution of holes from Cu to O therefore signals an increase in Cu–O bond covalence, i.e., a decrease in the charge-transfer gap and an increased contribution of O to unoccupied bands and of Cu to occupied bands. The concurrent increase in *T*_c, max_ under pressure is consistent with the proportionality between *T*_c, max_ and the O hole content seen by NMR. Studies using other methods also suggest an increasing *T*_c, max_ with a decreasing charge-transfer gap (26–28). Recently, Kowalski et al. (5) solved the three-band Hubbard model with parameters that capture the variable charge-transfer gap and bond covalence. Their results reproduce the varying, material-dependent O hole contents found with NMR (3) that scale with the maximum *T*_c_ (4). Kowalski et al. also find that the optimal doping level increases with decreasing charge-transfer gap and increasing *T*_c, max_, which, interestingly, fits the trend of mismatching domes in Fig. 2*B*.

Our results suggest that the sought-after intrinsic effect of pressure on *T*_c_, cf. **Eq. 2**, is a decrease of the charge-transfer gap, i.e., an increase in planar Cu–O bond covalence. Although our sample set and pressure range were limited, a simple estimate for the necessary changes of the planar charge contents under pressure in underdoped YBa_2_Cu_3_O_6.63_ shows quantitative agreement with the changes in planar charges we find. Also, Sadewasser et al. (29) estimated for the intrinsic pressure effect on *T*_c_ in YBCO about 0.1 K/kbar. The data here show an increase of 2*n*_O_ under pressure for all samples of about 0.042(6)%/kbar, cf. Fig. 5*C*. When multiplied with the slope of the *T*_c, max_/(2*n*_O_)≈ 200 K/hole, this gives 0.084(1) K/kbar, in good agreement with (29).

While our results qualitatively and quantitatively account for the intrinsic pressure effect that increases *T*_c, max_ in YBCO, the pressure phenomenology of *T*_c_ differs somewhat for different cuprate families. Clearly, the specific crystal structure and doping level should have an influence on how much pressure affects doping and changes planar bonding.

For La_2 − x_Sr_x_CuO_4_, for instance, *T*_c_ increases with pressure for all doping levels, indicating that pressure causes an intraplanar charge redistribution that increases (decreases) planar O (Cu) hole content and has hardly any effect on doping. The latter is also consistent with the pressure-independent Hall coefficient for all doping levels of this family (11).

For the Bi-, Tl- and Hg-based cuprate materials that can be realized in single-layer as well as different multilayer configurations, the pressure phenomenology is much more complex, e.g., including nonmonotonic *T*_c_-dependence on pressure for some materials. However, an interesting question concerns triple-layer materials (and beyond), as these exhibit distinct outer and inner CuO_2_ layers and, under pressure, can exhibit two maxima in *T*_c_. Perhaps, this relates to different effects of pressure on the different layers in terms of intraplanar and interplanar charge distribution as well as doping. The latter effect has already been indicated by first-principle calculations (22).

The weak *T*_c_-dependence on pressure in optimally electron-doped materials (30–32) could be accounted for by similar effects as in YBCO, i.e., compensating effects on *T*_c_ with pressure increasing *T*_c, max_ while also pushing the system to the underdoped regime through hole doping.

Finally, we would like to emphasize that both the previously reported proportionality between *T*_c, max_ and planar O hole content for different cuprate families (3, 4) and the increase of *T*_c, max_ under pressure by increasing planar O hole content reported here do not give any explanation for the peak of *T*_c_ at optimal doping and the superconducting dome. Only the height of the latter, *T*_c, max_, as well as other cuprate properties (33) appear to be fundamentally linked to the role of O in the planar structure.

The role of O holes measured by NMR (3, 4, 34) that reflect the bond covalence and the charge-transfer gap has to be of crucial importance for material chemistry as well as any theoretical attempt at understanding cuprate superconductivity. Mounting evidence, from other probes (6, 27) as well as theory (5, 26, 28, 35), also points to the significance of the charge-transfer energy for cuprate superconductivity.

## Materials and Methods

### A. Sample Preparation.

High-quality single crystals of YBCO were grown in nonreactive BaZrO_3_ crucibles and annealed as described elsewhere (36). The resulting fully oxygenated single crystals (y = 1) were twinned within the a–b plane. For the Y-6.9 sample, a microcrystal of an approximate size of 150 × 100 × 100 μm^3^ was cut from the slab and subsequently ^17^O exchanged, as previously described in ref. (20), which results in nearly optimally doped YBCO. In order to produce the ^17^O-enriched underdoped samples Y-6.5 and Y-6.85, we exchanged larger single crystals with ^17^O and subsequently annealed them to obtain the desired chain O content. They were cut into microcrystals afterward.

Prior to inserting the crystals into the pressure cell, the crystal axes were determined by polarized light that can easily identify domain boundaries in the twinned *a*–*b* plane at the surface. The crystals were fixed to one of the culet surfaces with epoxy so that the *c* axis is nearly parallel to the culet surface (*SI Appendix*, Fig. S4*A*). After closing the pressure cell, the *T*_c_ of the enclosed sample was determined using an NMR probe with a cryostat in zero field. The circuit was tuned at about 200 MHz at a temperature slightly above *T*_c_. Then, the temperature was lowered throughout the superconducting transition, and the concomitant change of the tank circuit frequency was monitored; the process was repeated by starting below *T*_c_ and raising the temperature. *T*_c_ was defined as the upper temperature where about 10% of the rapid frequency shift had occurred (*SI Appendix*, Fig. S5).

### B. Pressure Cell Preparation.

Our home-built pressure cells have cylindrical cell bodies with a diameter of about 17 mm and a height of about 20 mm (*SI Appendix*, Fig. S1*A*). The cell body is made from titanium. Optical access to the sample region is possible due to transparent anvils (along the cell axis) and 3 drilled holes in the cell body in the radial direction at angles of 120°. The latter allow for an inspection of the anvils and the gasket while the cell is closed to avoid destruction of the single crystal. The ruby luminescence technique was used to measure the pressure through the axial hole (37). Further details on the preparation of the cell, including the gasket, can be found elsewhere (38).

### C. NMR Experiments.

For the experiments, commercial Bruker or Tecmag pulse spectrometers were used with 11.7-T or 17.6-T superconducting magnets. The anvil cells were mounted on regular homemade probes that fit commercial cryostats for temperature variation. Spin echo (*π*/2 − *τ* − *π*) pulse sequences were employed, and if possible, whole transitions were excited and recorded, while frequency stepped echoes were employed for broad lines. The *π*/2 pulse length for a typical experiment was accordingly 0.5 μs or 7 μs. The average pulse power varied between 10 mW and 5 W (note that the small volume of the RF microcoils requires rather low power levels).

Different RF microcoil designs were tested, with various filling and Q factors, according to different sizes and shapes of the crystals. The microcoils were wound from an insulated silver wire (Goodfellow Cambridge Ltd.) with a diameter of 25 μm (5 μm insulation). The DC resistances measured on the closed cells were found to vary between ∼0.7*Ω* and ∼ <  *SPSDOUBLEDOLLAR* >  1.5*Ω* at room temperature (the lead resistances are smaller due to a larger diameter). With a typical coil inductance of 50 nH, this is in agreement with the measured Q factors that ranged between 20 and 40 (the RF skin depth is similar to the radius of the wire).

For the first cell (Y-6.5 crystal), we used microcoil with nearly elliptical cross-section to increase the filling factor. The crystal itself was extremely flat and small. It had the dimensions of approximately 90 × 90 × 40 μm^3^. The filling factor was about 0.13 (*SI Appendix*, Fig. S3*B*).

For the second cell (Y-6.85 crystal), a double-wound microcoil was used with a higher inductance and greater mechanical stability *SI Appendix*, Fig. S3*A*. The dimension of the crystal was 140 × 140 × 90 μm^3^. The filling factor of this coil was about 0.3.

For the third cell (Y-6.9 crystal), a regular cylindrical coil was used. The crystal had the dimensions 150 × 100 × 100 μm^3^. The filling factor of the coil was estimated to be about 0.4.

Since the signal-to-noise ratio (SNR) is critical, the noise was always measured and verified that it is of thermal origin, predominantly from the RF microcoil (an overall noise figure of about 1.25 dB was determined at room temperature).

The highest SNR (per scan) measured (in the time domain) on the central transition of planar ^63^Cu for *c* ∥ *B*_0_ at room temperature and a bandwidth of 5 MHz was *SNR* = 4.9 × 10^−2^ for the Y-6.9 cell; for the Y-6.85 and Y-6.5 cell, the SNR was about 3.2 × 10^−2^ and 0.4 × 10^−2^, respectively. For the planar O central transition, at a bandwidth of 2 MHz, we found SNRs of 2.9 × 10^−2^, 1.8 × 10^−2^, and 0.12 × 10^−2^ for Y-6.9, Y-6.85, and Y-6.5, respectively. With the necessary repetition times, a single spectrum could require 24 h of signal averaging. Due to the low signal (and SNR) for Y-6.5, only a limited set of data was recorded. Nutation experiments were performed to find the pulse lengths that were close (within factor of two) to the estimated RF amplitudes.

For the orientation of a cell with respect to the magnetic field *B*_0_, a goniometer that was mounted on the home-built NMR probe was used (*SI Appendix*, Figs. S1*B* and S2). While the single crystals were glued to one anvil with the *c*-axis parallel to its culet surface, the true crystal orientation was measured with the goniometer that holds the anvil cell (16). If the satellite linewidths and SNRs permitted, the satellite resonances were followed as a function of angles, cf. ref. (20). Otherwise, angular dependences for the planar Cu central transition were recorded.

The full angular dependence of the Cu NMR central transition of the Y-6.5 cell is shown in *SI Appendix*, Fig. S4*B*.

### D. Modes of Chemical Doping and Stoichiometry.

The chemical modifications to achieve doping in different cuprate families can differ significantly and therefore can relate to very different rates of doping of the CuO_2_-plane. In this manuscript, we adopt the already previously used notation (3) to reflect this in the stoichiometry, e.g., in the legend of Fig. 2.

We use “x,” typically x ∈ (0,0.3), where doping is achieved by partial cation substitution in the charge reservoir layer by a different valence. This should correspond directly to planar doping, e.g., doping *x* = x in hole-doped La_2 − x_Sr_x_CuO_4_; or *x* = −x in electron-doped Pr_2 − x_Ce_x_CuO_4_, which is borne out by NMR results (*ζ* ≈ x) without any adjusted parameters (3)

We use “y” ∈ (0,1) for the occupation level of the chain oxygen site in RE-123 compounds like YBCO. Here, the added chain O is nominally −2, i.e., “donating 2 holes.” But, also the corresponding chain Cu changes from nominally +2 to +1, and we have two CuO_2_-planes per unit cell, such that in the first approximation, one may expect *x* ≈ 0.5 ⋅ y. However, other valences in the charge reservoir can be expected to change as well.

We use δ, typically only a few %, in the formula for cuprate materials where interstitial O content in nonstoichiometric sites controls doping. This is found in Hg-, Bi-, and Tl-based cuprates that can also be realized in different multilayer configurations. Here, barring valence changes in the charge reservoir, the naive expectation would be that the interstitial O takes two electrons, such that stoichiometry suggests *x* ≈ 2 ⋅ δ.

### E. Determination of Charges.

The Cu and O splittings along the respective principle axes are related to the planar hole densities as follows (3, 34):

[3] $$\begin{array}{cc} {}^{17}\nu_{Q,\sigma } & =2.45\;\mathrm{MHz}\cdot n_{O}+0.39\;\mathrm{MHz}. \end{array}$$

[4] $$\begin{array}{cc} {}^{63}\nu_{Q,c} & =94.3\;\mathrm{MHz}\cdot n_{\mathrm{Cu}}-5.68\;\mathrm{MHz}\cdot \left(8-4n_{O}\right). \end{array}$$

In the case of the Y-6.5 sample, only splittings in *c*-direction could be measured, where the changes of the splitting are only half of what is observed along the bond, i.e., *Δ*_*p*_^17^*ν*_Q, c_ = 2.45 MHz/2 ⋅ *Δ*_*p*_*n*_O_.

In order to determine *n*_O_ from ^17^*ν*_Q, σ_ for the initial chemical doping level for this sample, we took literature data summarized in ref. (34) on ^17^*ν*_Q, c_, ^17^*ν*_Q, σ_, *T*_c_ and O content for various doping levels of YBa_2_Cu_3_O_6 + *y*_.

## Supplementary Material

Appendix 01 (PDF)

## Data Availability

All study data are included in the article and/or *SI Appendix*.

## Footnotes

^*^The degree of sharing of the inherent hole, nominally on Cu, and the distribution of chemically doped charges both reflect contributions of Cu and O orbitals to occupied and unoccupied electronic states. In a chemistry context, this can be said to reflect the planar Cu–O bond covalence; the higher the sharing, the higher the covalence is. In the context of prevailing models for cuprate physics, a higher O hole content reflects a smaller charge-transfer gap. For the purposes of this manuscript, we consider both statements to be equivalent.
