# Supplementary material for: How pressure enhances the critical temperature of superconductivity in YBa2Cu3O6+y
Source: Proc Natl Acad Sci U S A. 2023 Jan 6;120(2):e2215458120. doi: 10.1073/pnas.2215458120 (PMC9926205; doi:10.1073/pnas.2215458120)
Supplement: Supplementary file 1 — Appendix 01 (PDF) [file pnas.2215458120.sapp.pdf]

Supporting Information for

How pressure enhances T_c of high temperature superconductor $YBa_2Cu_3O_{6+y}$

Michael Jurkutat, Carsten Kattinger, Stefan Tsankov, Richard Reznicek, Andreas Erb and Jürgen Haase

Corresponding Author Michael Jurkutat
E-mail: m.jurkutat@gmail.com
Corresponding Author Jürgen Haase
E-mail: j.haase@physik.uni-leipzig.de

This PDF file includes:

Supporting text
Figs. S1 to S9

Supporting Information Text

This supplementary information gives additional data plots and photographs for "How pressure enhances T_c of high temperature superconductor $YBa_2Cu_3O_{6+y}$". In particular information about the high-pressure NMR cells used, the preparation and handling of the microscopic single-crystals, and the orientation of the crystals within the cells in the field. Furthermore, a sample curve is shown which was used to determine $T_c(p)$. Also the temperature dependence of the RF-noise is presented as well as some NMR spectra.

1. High-pressure NMR cells used

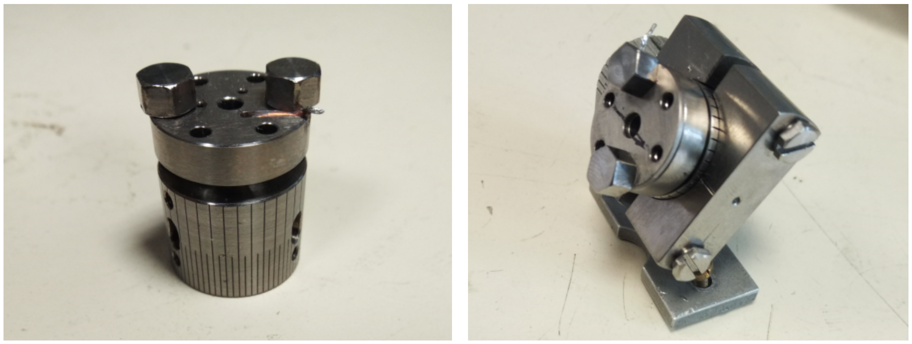

Fig. S1. *left*: Picture of the closed Y-6.5 cell. *right*: The Y-6.85 cell in a titanium goniometer.

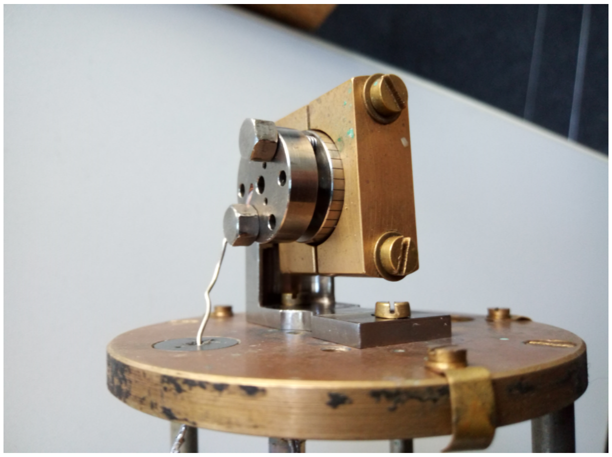

Fig. S2. Y-6.5 cell in two-axis brass goniometer mounted the homebuilt NMR probe.

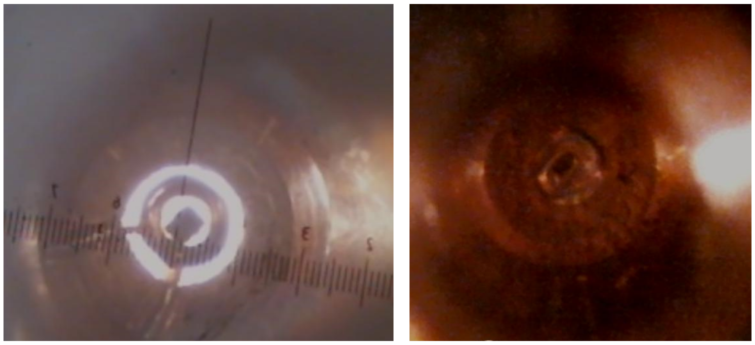

(a) Y-6.85 cell

(b) Y-6.5 cell

Fig. S3. *left*: Y-6.85 cell - picture of the sample chamber through the anvils at 5 GPa, one can see the doubly wound coil and the crystal inside. (b)Y-6.55 cell - picture of the sample chamber trough the anvils at 4 GPa, one can see the elliptically wound coil and the crystal inside.

2. Single Crystal

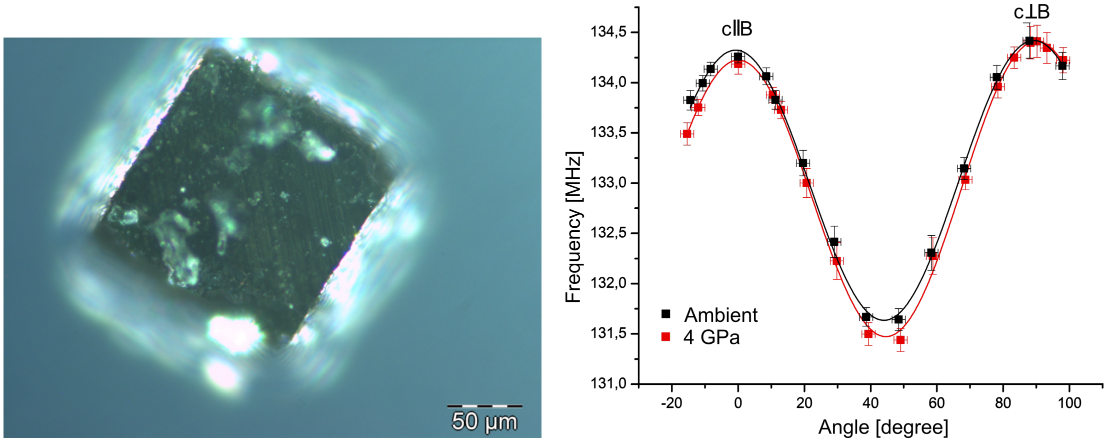

(a) Y-6.85 under polarized light

(b) Orientation dependent ^{63}Cu NMR

Fig. S4. (a) Y-6.85 under polarized light, one can see the twining lines (almost vertical) that reflect domain boundaries. (b) Y-6.5 Angular dependence of the ^{63}Cu central transition frequency at different pressure levels. The quadrupole and the Larmor freqencies at ambient conditions are found to be $\nu_{\mathrm{Q}} = 29.43\,\mathrm{MHz}$ and $\nu_{\mathrm{L}} = 133.58\,\mathrm{MHz}$. At $4\,\mathrm{GPa}$ these are changed to $\nu_{\mathrm{Q}} = 30.01\,\mathrm{MHz}$ and $\nu_{\mathrm{L}} = 133.51\,\mathrm{MHz}$.

3. T_{c} determination

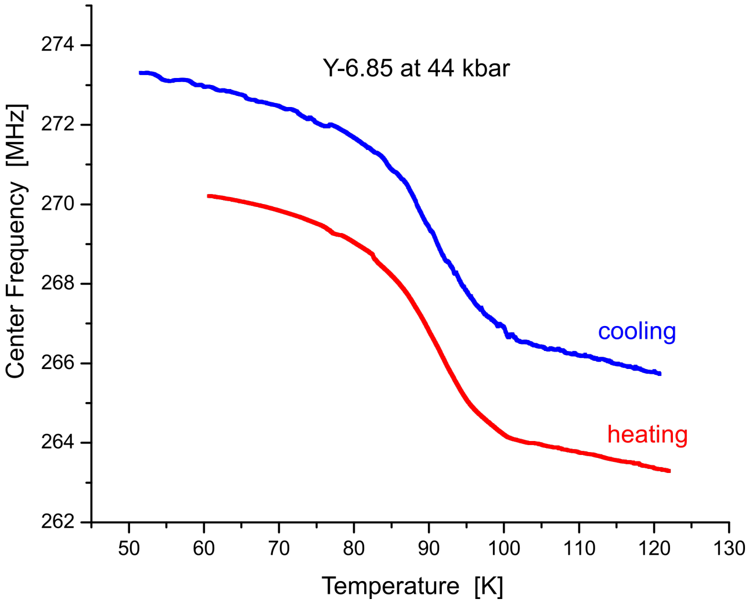

Fig. S5. Measurement of the center frequency of the resonance circuit vs. temperature at 44kbar in the y-6.85 cell for cooling and heating across T_c. One can see the change in the coils inductance due to the onset of the diamagnetic response in the superconducting transition.

4. Temperature dependence of the Noise

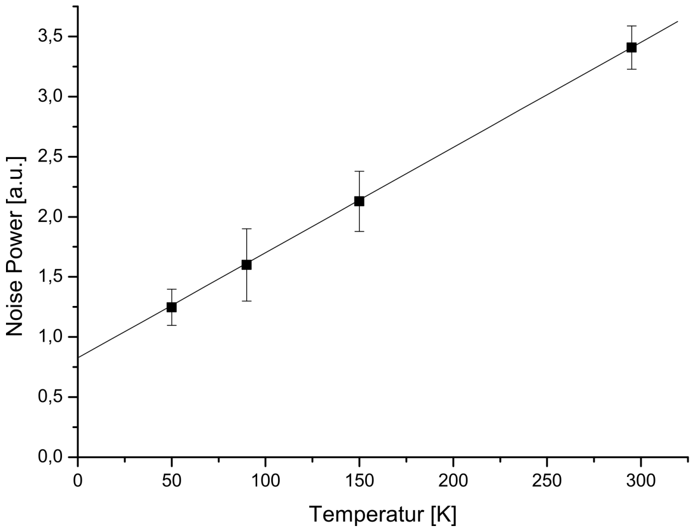

Fig. S6. Noise vs. temperature in Y-6.85 cell: The main noise source is the micro coil which is reduced linearly with temperature. This is expected from the thermal noise in a wire, the constant offset is due to the rest of the setup, particularly the preamp. The noise figure was about $1.25\,\mathrm{dB}$ at room temperature.

5. High-pressure NMR spectra

In order to determine the charge distribution, the orientation dependent quadrupole splitting for both planar nuclei had to be measured. Examples of typical spectra for both nuclei in all tree samples at different pressures are shown in Fig. S7.

Spectra of ^{17}O (spin $I = 5/2$) show the expected quintuplet of resonances with the central transition (CT) $(+1/2 \leftrightarrow -1/2)$ and four satellite transitions $(\pm 3/2 \leftrightarrow \pm 1/2,\ \pm 5/2 \leftrightarrow \pm 3/2)$, of which we only show two resonance lines in Fig. S7 A-F. (Examples of full spectra for both field orientations are given in Figs. S8 and S9.) The equidistant quadrupole splitting of the ^{17}O satellites is measured and the corresponding lineshapes give a histogram of the charge variation.

For planar ^{63}Cu ($I = 3/2$, three resonance lines) we show the quadrupole splitting only between the central transition Fig. S7 (G-I) and the upper satellite (J-L). The central transitions for all three samples have a similar resonance frequency at ambient pressure, but lower doped samples Y-6.5 and Y-6.85 show broader satellite lines. Under pressure all samples show decreasing NMR shift, evidenced by a decreasing CT resonance frequency, as well as line broadening. The upper ^{63}Cu satellites of Y-6.85 and Y-6.9 are very different from one another, despite similar doping, T_c, ^{17}O splittings and ^{63}Cu central transitions. The satellite of Y-6.9 shows a higher quadrupole splitting and, more strikingly, a sharp linewidth almost an order of magnitude smaller than that of Y-6.85. Due to too small SNR for Y-6.5, the ^{63}Cu quadrupole splitting and linewidth for this sample had to be determined from angular-dependent second-order effects on the CT frequency, and the calculated satellite frequency and estimated linewidth are indicated in red. Cu satellite linewidths of a few MHz are actually most typical in cuprates and only certain, well-ordered YBCO-configurations (, that also show the ^{17}O double peaks) show satellites less than 0.5 MHz wide. Similar to the ^{17}O double-peak phenomenon, commensurate vs. incommensurate charge density variations can explain these effects on ^{63}Cu linewidths.

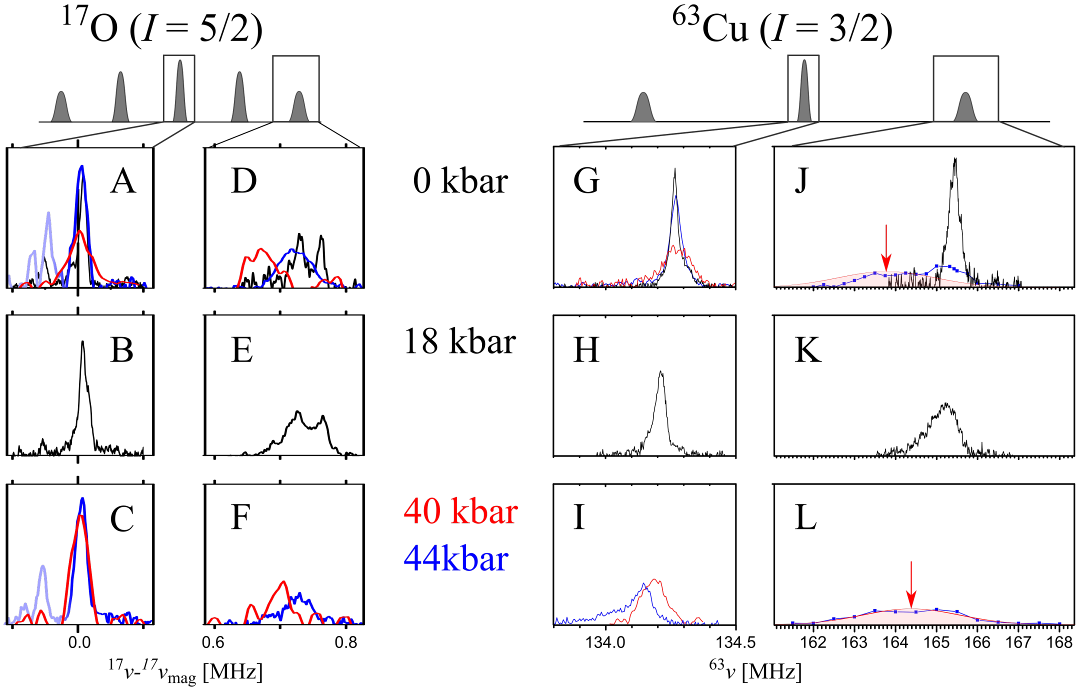

Fig. S7. Examples of room-temperature NMR spectra from the CuO_2 plane at different hydrostatic pressures. Planar ^{17}O ($I = 5/2$) and ^{63}Cu ($I = 3/2$) display, respectively, three-fold and five-fold quadrupolar-split spectra, see sketches at the *top*. For planar ^{17}O we show A - C the central transitions and D - F uppermost satellites (with magnetic frequency subtracted) and for planar ^{63}Cu G - I the central transitions and J - L upper satellites. Data for all three samples are shown: Y-6.9 (black) shows characteristic ^{17}O double peak satellites (D and E) and rather sharp Cu lines (G, H, J and K). Y-6.85 (blue) shows similar ^{17}O satellite widths as Y-6.9, but without clear double peak features. Its Cu central transition is only slighty broader than that of Y-6.9 (G), but the satellites are about one order of magnitude broader (J). For Y-6.5 (red), due to weak signal (see Methods), ^{17}O had to be measured at higher field and for ^{63}Cu the quadrupole frequency had be determined from the angular dependent frequency of the central transition and the resulting satellite frequencies are indicated by a red arrow and approximate linewidths (shaded red) estimated from second order quadrupole broadening of the central transition.

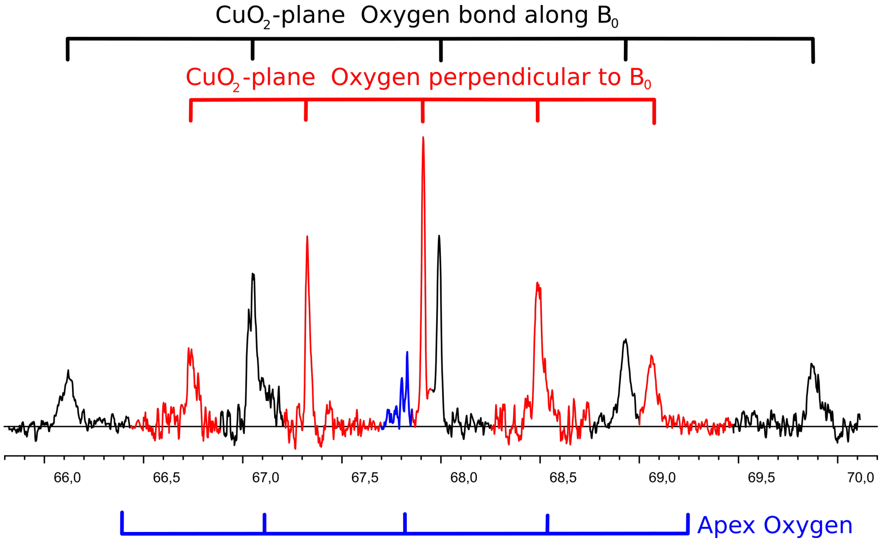

Fig. S8. Y-6.85: Oxygen spectrum in $ab \parallel B_0$ at room-temperature. Marked in black is the spectrum of the half of the planar O sites with the their planar bond along B_0, in red is the spectrum of the other half of O sites with their σ-bond perpendicular to B_0. Marked in blue is the central transition of the apex oxygen cite, the satellite transitions of apex site are not resolved here.

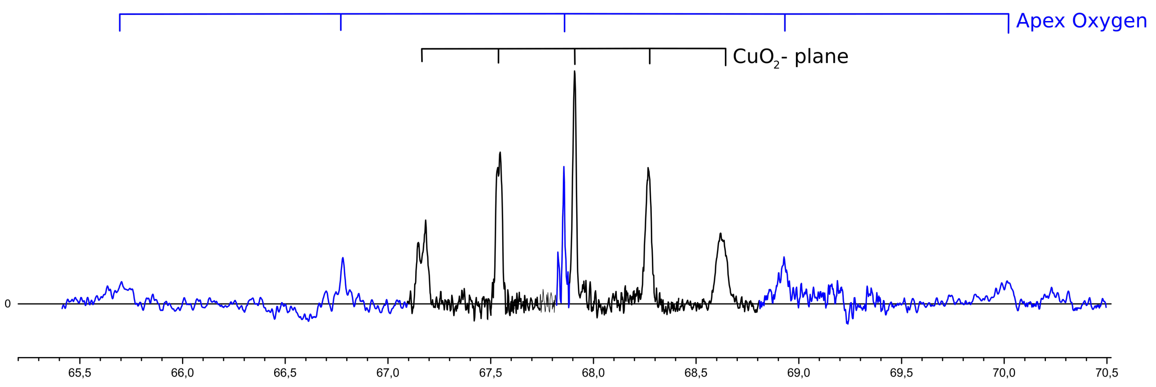

Fig. S9. Y-6.85: Oxygen spectrum at room-temperature in $c \parallel B_0$. Marked in black is the planar O site, in blue the apex O.
